# Hydrogen sulfide combined with biomaterials for wound healing: a bibliometric analysis

**DOI:** 10.4103/mgr.MEDGASRES-D-26-00152

**Published:** 2026-09-12

**Authors:** Mengyue Du, Huanhuan Wang, Xiping Zhang, Shuqin Li, Sihan Cao, Chang Yao

**Affiliations:** Department of Breast Surgery, Affiliated Hospital of Nanjing University of Chinese Medicine, Nanjing, Jiangsu Province, China

**Keywords:** chronic wounds, controlled release, gas therapy, gasotransmitter, hydrogel, nanomaterials, scaffolds, tissue regeneration, wound microenvironment, wound repair

## Abstract

Hydrogen sulfide (H_2_S) is an extensively multifunctional gasotransmitter that plays a critical role in wound healing. Due to advances in biomaterials science, H_2_S can be incorporated into biomaterial platforms for wound healing applications. However, research in this field remains fragmented, and a systematic analysis of publication trends, collaboration networks, and thematic evolution is still lacking. In our study, we used a bibliometric approach to investigate the development and evolution of this field. A total of 116 publications from 2005 to March 31, 2026 were collected and analyzed using VOSviewer software and the Bibliometrix R package. Our findings showed that (1) the first relevant publication appeared in 2015; the number of annual publications was low at the beginning and increased gradually, with a clear acceleration after 2022; (2) China was the leading country in terms of publication output, followed by the United States, and the most productive institutions were Wenzhou Medical University and Shanghai Jiao Tong University; and (3) keyword analysis showed that “hydrogen sulfide,” “wound healing,” and “hydrogel” were the core research themes; current research hotspots include “anti-inflammatory,” “antibacterial,” “pH responsive,” “angiogenesis,” and “multimodal therapy,” while “bacterial ferroptosis” has emerged as a frontier topic. Overall, H_2_S in combination with biomaterials constitutes a rapidly developing interdisciplinary area of research. Further investigations should focus on emerging mechanisms such as bacterial ferroptosis, the development of intelligent multi-responsive biomaterials, and multimodal therapies that involve H_2_S together with other therapeutic modalities to facilitate the translation of laboratory findings into clinical applications.

## Graphical abstract

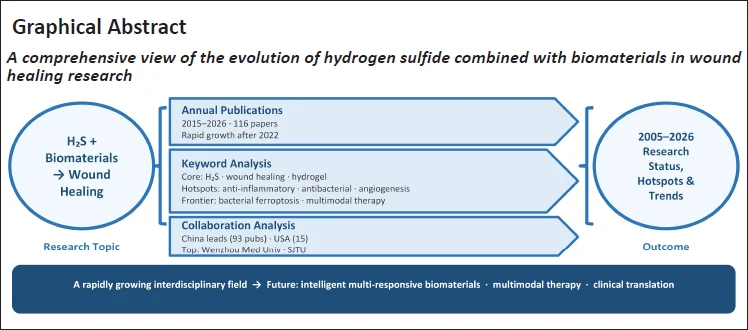

## Introduction

Chronic wounds, including diabetic ulcers, pressure injuries, and burns, are characterized by chronic inflammation, oxidative stress, poor angiogenesis, and the risk of infections, making wound recovery very difficult.^1^ While some success has been reported with the use of traditional wound dressings and growth factors, there are limitations related to bioavailability, degradation, and a lack of multi-targeting ability in these treatments.^2^,^3^

Research involving gas signaling molecules in wound healing is a recent trend due to their numerous roles in modulating the inflammatory response, antibacterial activity, and angiogenesis.^4^,^5^ Concerning angiogenesis, hydrogen sulfide (H_2_S) can control the expression of vascular endothelial growth factor through the stimulation of the phosphatidylinositol 3kinase/protein kinase B (Akt) and mitogenactivated protein kinase/extracellular signalregulated kinase pathways, resulting in endothelial cell proliferation, migration, and tubule formation.^6^,^7^ As for the regulation of inflammation, H_2_S inhibits nuclear factor-κB signaling, decreasing the production of the pro-inflammatory cytokines tumor necrosis factor-α and interleukin-6 and shifting macrophage polarization from the pro-inflammatory type (M1) to the pro-regenerative type (M2).^8^,^9^,^10^ Regarding the role of H_2_S in oxidative stress, H_2_S acts as a free radical scavenger and promotes increased expression of endogenous antioxidant enzymes such as superoxide dismutase and glutathione peroxidase through the nuclear factor erythroid 2related factor 2 pathway.^11^ Additionally, H_2_S has a broad spectrum of antibacterial properties due to the disruption of bacterial cell membrane structure and metabolism.^12^,^13^ Therefore, H_2_S can be regarded as a multi-target therapy, and positive outcomes have already been achieved in animal models of diabetic wounds, infected wounds, and burns.^9^,^14^,^15^

Nonetheless, due to its extremely short half-life, rapid diffusion, unpredictable release, and toxic nature at higher concentrations, the use of H_2_S in clinics is still quite limited.^16^,^17^ Biomaterial carriers, such as hydrogels, nanoparticles, electrospun fibers, and microneedle patches, guarantee the local delivery of H_2_S, resulting in decreased toxicity.^18^,^19^,^20^ Owing to the developments in the field of biomaterials engineering, H_2_S delivery systems are moving from sustained-release systems to intelligent and stimuli-responsive systems, allowing the delivery of H_2_S in response to changes in pH, reactive oxygen species (ROS), glucose, enzymes, and light stimuli.^21^,^22^,^23^ Moreover, multifunctional platforms are being developed that provide simultaneous delivery of H_2_S, bacterial inhibition, immunomodulation, angiogenesis promotion, and wound microenvironment remodeling.^24^

We conducted a bibliometric analysis of relevant literature published between 2005 and March 31, 2026, and aimed to map of the knowledge structure and developmental pathway of the H_2_S and biomaterials for wound healing, identify hot topics and future research directions, and provide evidence-based references for future research and the translation of research into clinical applications.

## Methods

### Literature search

The literature review was conducted using three bibliographic databases, namely Web of Science Core Collection, PubMed, and Scopus. The time period for the literature review was from January 1, 2005, to March 31, 2026. The search strategy consisted of combining three conceptual groups of terms: (1) H_2_S and its donors, (2) biomaterials, and (3) wound healing and tissue repair. The search strategies are shown in **Table 1** and **Additional file 1**.

**Table 1:** Literature search strategy

Conceptual group	Key terms	WoS field	Scopus field	PubMed field
Hydrogen sulfide & donors	Hydrogen sulfide, H_2_S, sodium hydrosulfide, GYY4137, diallyl trisulfide, H_2_S donor, etc.	TS	TITLE-ABS-KEY	[Title/Abstract]
Biomaterials	Hydrogel, scaffold, nanoparticle, dressing, nanocomposite, microneedle, etc.	ALL	TITLE-ABS-KEY	[Title/Abstract]
Wound healing & tissue repair	Wound heal, tissue engineering, chronic wound, diabetic ulcer, implant infection, etc.	ALL	TITLE-ABS-KEY	[Title/Abstract]

WoS: Web of Science Core Collection.

### Inclusion and exclusion criteria

The inclusion criteria were as follows: (1) studies dealing with the use of H_2_S in combination with biomaterials in wound healing; (2) journal articles or reviews; (3) publications written in English; and (4) journal articles published between January 1, 2005 and March 31, 2026, or made available online ahead of print during this period.

The exclusion criteria comprised: (1) duplicate papers; (2) conference proceedings, abstracts, books, book chapters, and retracted papers; and (3) studies irrelevant to the research problem.

### Data collection and processing

As a result of the preliminary search, a total of 692 records were retrieved (Web of Science: 224; Scopus: 345; PubMed: 123). After merging and deduplicating the records using Zotero (version 9.0; Corporation for Digital Scholarship, Vienna, VA, USA), 411 unique records remained. After title, abstract, and full-text screening according to the inclusion criteria, 116 publications remained in the final dataset, including 103 original articles and 13 reviews (**Figure 1**).

**Figure 1:**
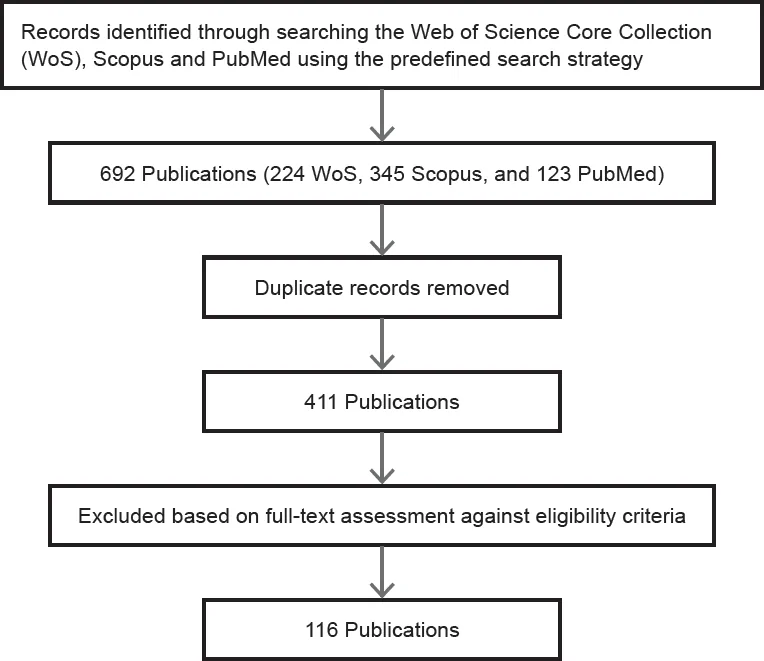
Flow chart of the literature screening process.

Before exporting the data, the database provenance of the 116 publications in the dataset was considered. All records retrieved from PubMed were found to be indexed in the Web of Science or Scopus databases. Since Web of Science and Scopus provided broader coverage of bibliometric fields, PubMed records were not used as an independent source for the analysis. Therefore, the final dataset for the analysis was compiled from Web of Science and Scopus records.

In order to conduct the bibliometric analysis, the Web of Science records were exported in plain-text format, whereas the Scopus records were exported in CSV format. Both files were merged and analyzed in R using the Bibliometrix package (version 4.1.4; https://www.bibliometrix.org).^25^ For visualization in VOSviewer, which requires files in plain-text format, the Scopus CSV records were manually converted into plain-text format, with field labels standardized according to the Web of Science format.

## Results

### Annual publication trends in H_2_S-biomaterial research for wound healing

The first papers appeared in 2015, which means that investigations into H_2_S effects along with biocompatible materials in relation to wound healing represent an emerging area of study. The output was low and sporadic from 2015 until 2019 (1–3 articles per year), whereas the output began to increase steadily in 2020 (*n* = 5). A significant increase has been observed since 2022 with 10 articles published, and the number of articles published increased to 17 in 2023 and to 25 in 2024. However, the peak was reached in 2025, with 30 articles. It is noteworthy that 16 papers were published in the first quarter of 2026 by March 31, 2026 (**Figure 2**).

**Figure 2:**
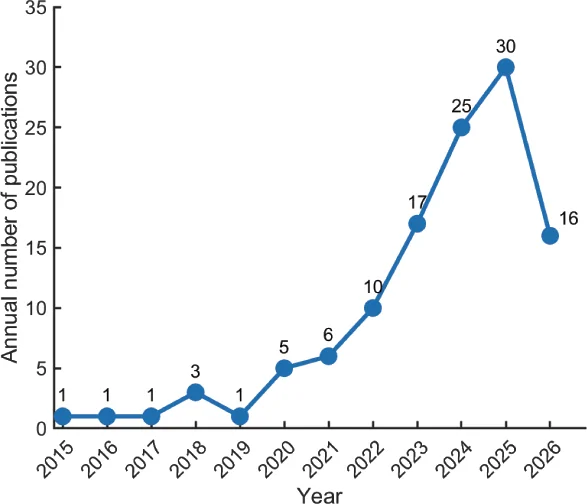
Annual publication trends in H_2_S-biomaterial research for wound healing (2015–March 31, 2026). Data for 2026 are partial, covering January 1 to March 31 only. Created with Bibliometrix R package. H_2_S: Hydrogen sulfide.

### Country and institutional publications and collaboration networks in H_2_S-biomaterial research for wound healing

As stated in **Table 2**, China ranked first in the world with 93 articles, while the United States ranked second with 15. The other countries with lower publication numbers, included Italy (4), India (3), Portugal (3), and the United Kingdom (3). The international co-authorship network found that China and the United States occupy central positions in the co-authorship network and have the strongest collaborative ties (**Figure 3A**). While China collaborates with several countries, including the United Kingdom, Australia, Belgium, Thailand, the Netherlands, and South Korea, the United States collaborates with the Netherlands, Saudi Arabia, South Korea, and Thailand. Conversely, there were some countries, such as India and Vietnam, that had not collaborated with any other country (total link strength = 0).

**Figure 3:**
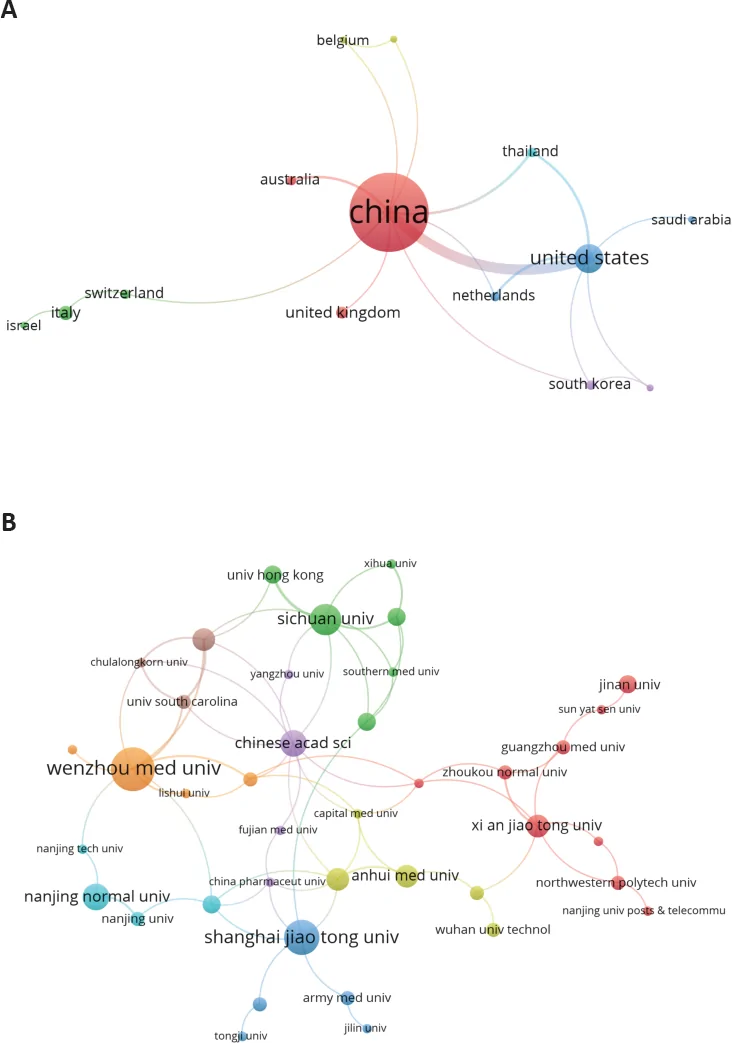
Collaboration networks of countries and institutions in H_2_S-biomaterial research for wound healing. (A) International collaboration network among countries. (B) Institutional collaboration network. Created with VOSviewer. H_2_S: Hydrogen sulfide.

**Table 2:** Top 13 countries/regions in H_2_S-biomaterial research for wound healing

Rank	Country	Documents	Citations	Total link strength
1	China	93	3195	19
2	United States	15	1021	16
3	Italy	4	123	2
4	India	3	29	0
5	Portugal	3	61	1
6	United Kingdom	3	61	1
7	Australia	2	35	2
8	France	2	12	1
9	Netherlands	2	17	3
10	South Korea	2	21	3
11	Switzerland	2	62	2
12	Thailand	2	361	4
13	Vietnam	2	3	0

H_2_S: Hydrogen sulfide.

From **Figure 3B** and **Table 3**, it can be seen that Wenzhou Medical University and Shanghai Jiao Tong University were the most prolific institutions, with 10 and 8 papers, respectively. As far as collaborative intensity is concerned, the core hubs of the network are Wenzhou Medical University and Sichuan University, with the highest total link strength of 12, followed by the Chinese Academy of Sciences and Washington State University, with a total link strength of 10 each. Interestingly, although Washington State University published only five papers, it received 546 citations and had strong collaborative connectivity, indicating relatively high citation visibility and collaborative centrality within this dataset.

**Table 3:** Top 14 institutions in H_2_S-biomaterial research for wound healing

Rank	Institution	Documents	Citations	Total link strength
1	Wenzhou Medical University	10	558	12
2	Shanghai Jiao Tong University	8	293	6
3	Sichuan University	7	445	12
4	Chinese Academy of Sciences	6	463	10
5	Nanjing Normal University	6	92	3
6	Anhui Medical University	5	63	4
7	University of Science and Technology of China	5	195	7
8	Washington State University	5	546	10
9	Xiʹan Jiaotong University	5	223	7
10	Jinan University	4	36	1
11	Southeast University	4	332	5
12	Southwest Jiaotong University	4	131	6
13	The University of Hong Kong	4	121	5
14	Zhejiang University	4	105	4

H_2_S: Hydrogen sulfide.

### Author collaboration in H_2_S-biomaterial research for wound healing

As shown in **Figure 4A**, regarding research output, Chen Y and Wang Y (11 documents each) were identified as the most productive authors, followed by Wang Z (10 documents). As depicted in **Figure 4B**, the co-authorship network included 63 authors, who were grouped into five main clusters, reflecting multiple closely collaborating research teams. Within this network, scholars such as Xu Ke and Li Yi acted as pivotal cross-cluster bridges that effectively integrated independent research subgroups and promoted collaboration.

**Figure 4:**
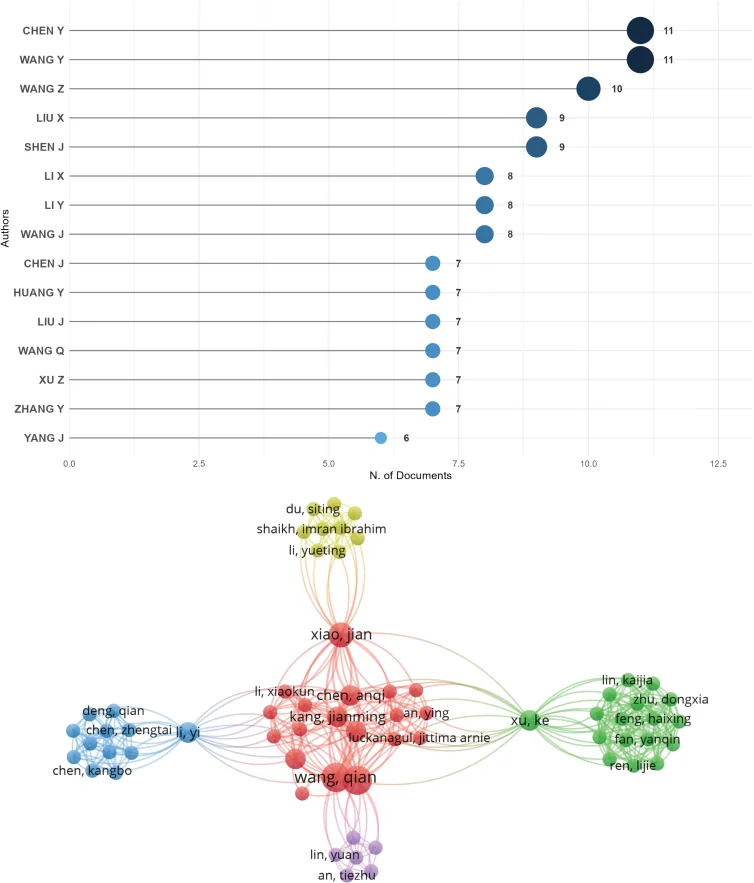
Core authors and collaboration network in H_2_S-biomaterial research for wound healing. (A) Most productive authors. (B) Author collaboration network. Created with VOSviewer and Bibliometrix R package. H_2_S: Hydrogen sulfide.

### Core journals and cross-disciplinary collaboration network in H_2_S-biomaterial research for wound healing

**Figure 5** provides the distribution, trends over time, and citation network of the key journals in this subject area. The *Chemical Engineering Journal* was the most prolific journal (nine documents), followed by the *International Journal of Biological Macromolecules* (seven documents) and *ACS Applied Nano Materials*, *Acta Biomaterialia*, and *Biomaterials* (six documents each). From the trends over time, it is evident that there has been a significant increase in the number of documents published in the *Chemical Engineering Journal* and *International Journal of Biological Macromolecules* from 2023 onward. This makes both of these journal important sources of research outputs in this discipline. Moreover, the journal citation network reveals that *Biomaterials*, *Acta Biomaterialia*, and the *Chemical Engineering Journal* are the key journals.

**Figure 5:**
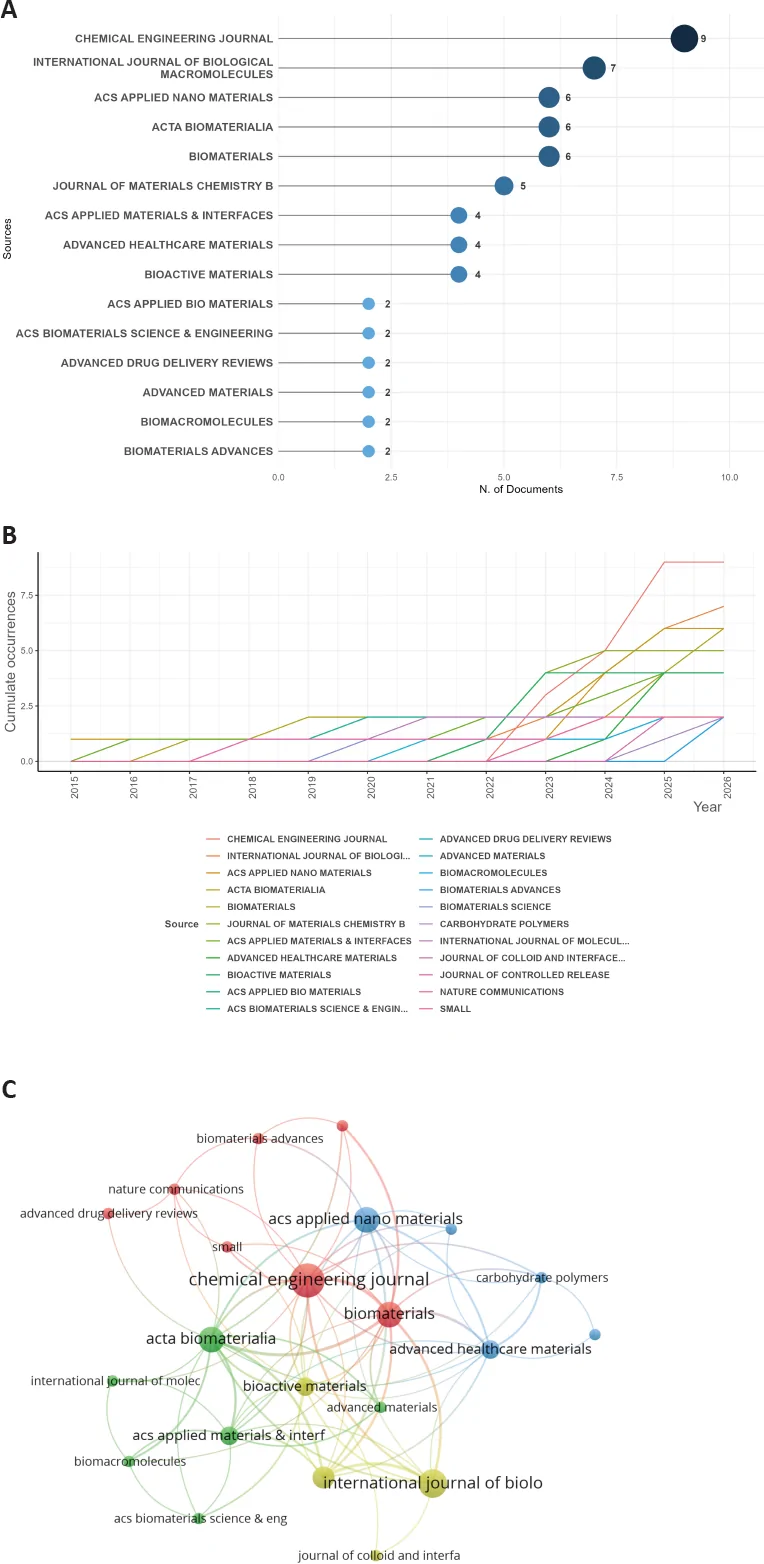
Publication distribution, temporal trends, and citation network of core journals in H_2_S-biomaterial research for wound healing. (A) Top journals by publication count. (B) Cumulative production of sources over time. (C) Journal citation network. Created with VOSviewer and Bibliometrix R package. H_2_S: Hydrogen sulfide.

### Keywords in H_2_S-biomaterial research for wound healing

From the keyword frequency distribution shown in **Figure 6**, high-frequency keywords show the use of H_2_S together with biomaterials in wound repair applications. The most commonly used keywords include “hydrogen sulfide” (46 times), “wound healing” (31 times), and “hydrogel” (22 times). From this, it is clear that studies focus on hydrogel-based H_2_S gas therapy. Moreover, other common keywords are “gas therapy,” “diabetic wound healing,” and “hydrogen sulfide release,” showing that hydrogel delivery systems target chronic diabetic wounds using gas therapy. Additionally, other common keywords are “antibacterial,” “angiogenesis,” and “anti-inflammatory.”

**Figure 6:**
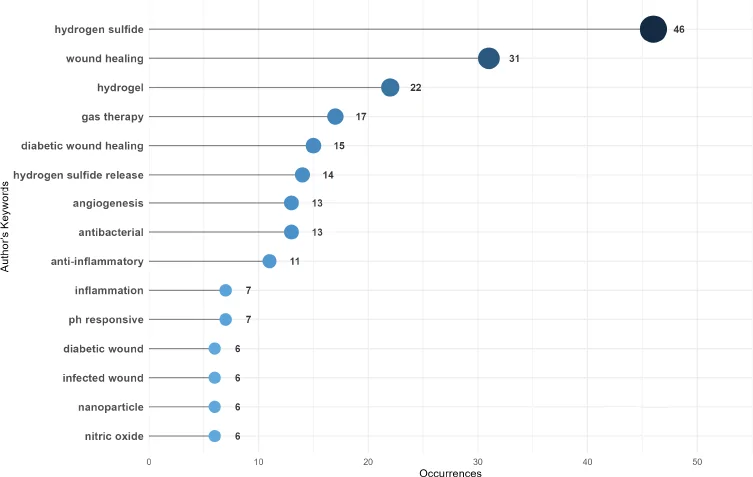
Frequency distribution of the most relevant keywords in H_2_S-biomaterial research for wound healing. Created with Bibliometrix R package. H_2_S: Hydrogen sulfide.

**Figure 7** provides an overview of the entire structure of topics and new trends in this domain based on the keyword density map and co-occurrence network. The keyword density map found H_2_S to be the dominant and most highly represented node, with nodes like wound healing, gas therapy, hydrogel, and diabetic wound healing existing around it. This shows that the dominant application of H_2_S-releasing hydrogels is in wound healing. The co-occurrence network has further classified these areas into clusters. The dominant clusters were found to include functional mechanisms, including anti-inflammatory properties and angiogenesis, and antibacterial mechanisms for diabetic wound healing. Apart from this, the existence of clusters for H_2_S release and drug delivery, along with relations to other gasotransmitters such as nitric oxide.

**Figure 7:**
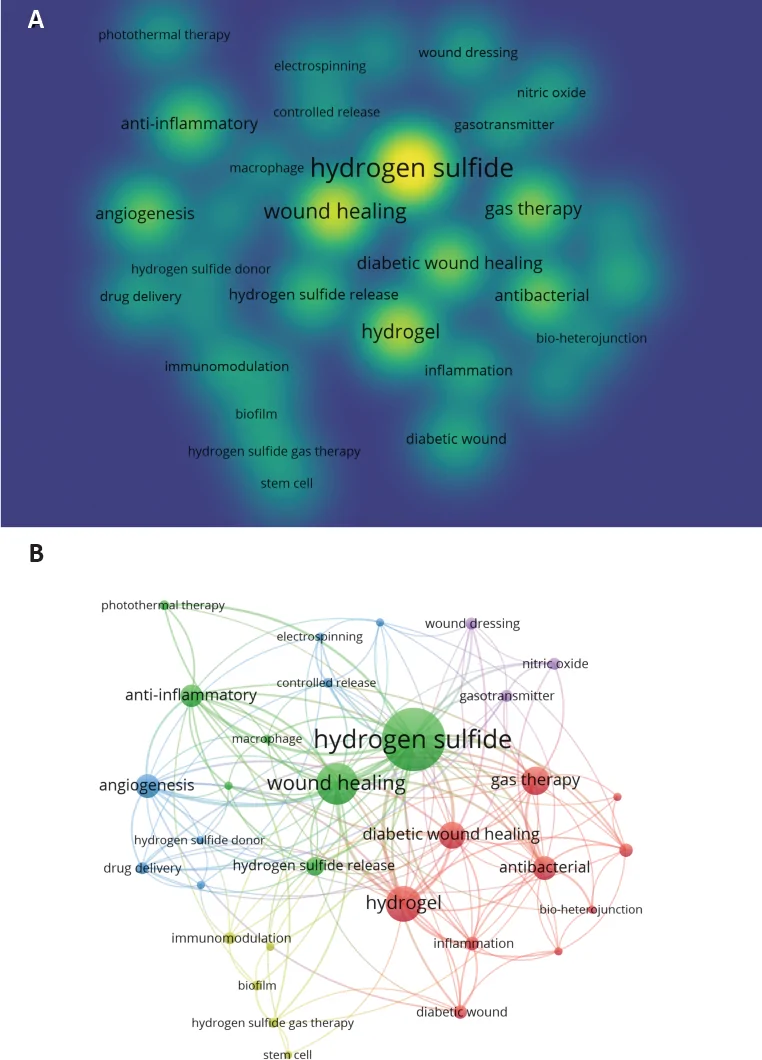
Keyword co-occurrence analysis in H_2_S-biomaterial research for wound healing. (A) Keyword density heatmap. (B) Keyword co-occurrence network. Created with VOSviewer. H_2_S: Hydrogen sulfide.

**Figure 8** provides the temporal trends in the research area. It begins with the use of conventional materials (such as electrospinning and silk fibroin), then moved on to the implementation of gasotransmitter strategies utilizing nitric oxide and carbon monoxide, and finally reached the use of hydrogels in the process of treating diabetic wounds and providing anti-inflammatory effects. Recent advancements in research have brought the wound microenvironment and ROS, along with the new idea of bacterial ferroptosis as a novel method for sterilization without the use of antibiotics.

**Figure 8:**
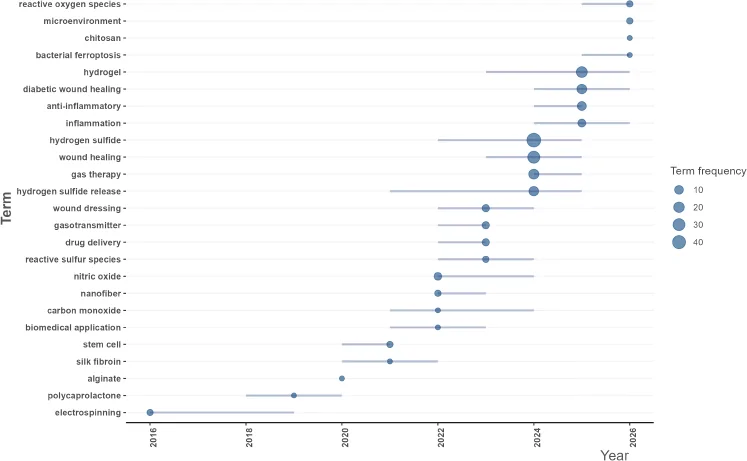
Temporal trends of keywords in H_2_S-biomaterial research for wound healing. Created with Bibliometrix R package. H_2_S: Hydrogen sulfide.

The strategic theme map reveals the dynamism with which the topics in this field and their development levels are changing. This map has divided keywords into four quadrants (**Figure 9**).

**Figure 9:**
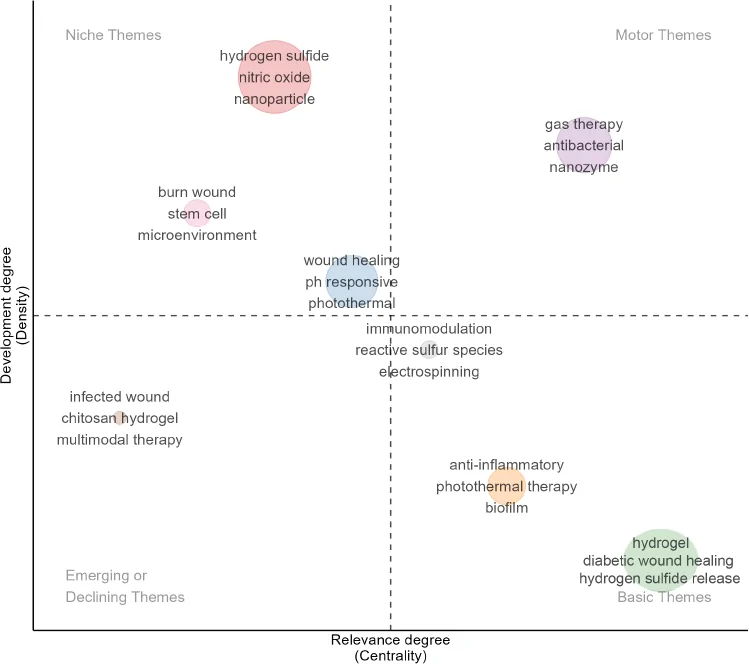
Strategic theme map in H_2_S-biomaterial research for wound healing. Created with Bibliometrix R package. H_2_S: Hydrogen sulfide.

(1) Basic themes quadrant: This quadrant features the most mature topics and some keywords like “hydrogel,” “diabetic wound healing,” “hydrogen sulfide release,” and “anti-inflammatory,” which form the basic foundation of this field of study. In addition, there are some other keywords like “immunomodulation,” “reactive sulfur species,” “electrospinning,” and “photothermal therapy,” which represent the broad range of materials science and biochemical knowledge used in the development of this field of study.

(2) Motor themes quadrant: This quadrant features “gas therapy,” “antibacterial,” and “nanozyme.” These are mature and highly topical topics that have served as the primary driving force behind the growth of this field.

(3) Niche themes quadrant: These themes are characterized by low centrality but high internal development density. The topics mainly include frontier issues, such as “hydrogen sulfide,” “nitric oxide,” “nanoparticle,” “burn wound,” “stem cell,” and “microenvironment.” This suggests that the underlying mechanisms and extended applications of gasotransmitters have established a substantial research foundation but have not yet been widely integrated into mainstream biomaterial applications.

(4) Emerging/declining themes quadrant: Both density and centrality are relatively low in this quadrant, mainly encompassing “infected wound,” “chitosan hydrogel,” and “multimodal therapy.” This quadrant typically comprises themes that are either in the early stages of interdisciplinary development or are established topics that are gradually moving away from the mainstream of the field.

## Discussion

### Overall findings and development trends

This study conducted a bibliometric analysis of 116 publications on “hydrogen sulfide combined with biomaterials for wound healing” identified through searches of the Web of Science Core Collection, PubMed, and Scopus databases from January 1, 2005, to March 31, 2026. The results indicate that research on H_2_S–biomaterial systems for wound healing is a relatively nascent field that has shown notable growth in publication activity in recent years, alongside increasing interdisciplinary integration and diversification of research topics. A notable observation is the late emergence of this research area. Although the search period spanned more than two decades, relevant publications did not appear until 2015. Since then, the number of yearly publications remained low in the early years, then gradually increased after 2020, and has accelerated markedly since 2022, implying growing interest in H_2_S-related uses in regenerative medicine.

These facts can be explained by the rising awareness of the potential of H_2_S as a therapeutic agent, with its anti-inflammatory, antioxidant, and antibacterial effects, together with progress in bioengineering to control gas delivery. Overall, all these findings indicate that this field has progressed rapidly to become an emerging field of study in regenerative medicine.

### National, institutional and author collaboration patterns

By country, China ranked first with 93 publications, and the USA ranked second with 15. China and the USA represent an efficient dual-core collaboration axis, which means that the development of H_2_S-biomaterials-based therapies relies on the collaborative efforts of these two countries. Moreover, it is worth mentioning that Thailand demonstrates a very high citation impact and a relatively low level of publication output at the same time, but it still has strong collaborative relationships with core countries. Hence, one should note that high-quality research in this narrowly specialized field is not always a product of high productivity, but sometimes a result of goal-oriented international collaboration. The lack of collaboration ties (total link strength = 0) indicates the fragmentation of this scientific field in its initial phase of development.

The institutions of China serve as collaboration hubs in this network, involving foreign universities, like Washington State University. As for Washington State University, powerful transnational ties could increase its academic impact despite the low number of publications. Therefore, international collaboration may be more important than publication count in determining academic impact.

Regarding the authors, it is notable that the analysis of the five co-authorship clusters reveals one peculiar characteristic of this field. Although the field has stable research teams, it requires several individuals who are able to play the role of “bridges” because they help to bypass possible academic barriers. In other words, the appearance of authors such as Xu Ke and Li Yi as cross-cluster authors indicates that collaboration among teams and methodological integration rely mostly on individual efforts in this field.

### Core journals and interdisciplinary knowledge flow

It is clear from the examination of source journals that there is a distinct interdisciplinary landscape comprising chemical engineering, biological macromolecules, and nanomaterials. The considerable influence of the *Chemical Engineering Journal*, the main source journal, as well as the highly productive *International Journal of Biological Macromolecules* and traditional journals focused on biomaterials (*Acta Biomaterialia* and *Biomaterials*), shows that materials engineering, chemistry, and biological sciences are strongly overlapping disciplines in this research area. From a temporal perspective, the emergence of the *Chemical Engineering Journal* and the *International Journal of Biological Macromolecules* after 2023 is an important milestone since it means that research on the topic has entered a fast-growing period in which research on H_2_S-based treatments has evolved from initial exploration into an emerging area. One should pay attention to the fact that this fast growth happens in the context of the development and perfection of hydrogel delivery systems for wound healing in cases of diabetes. Academically speaking, *Biomaterials*, *Acta Biomaterialia*, and *Chemical Engineering Journal* are the key network hubs as they have high centrality and connectivity and function as the leading sources of interdisciplinary communication. In addition, the connections between different journal clusters clearly indicate that the research has an interdisciplinary nature. The interdisciplinary fertilization of chemical engineering, polymer chemistry, and biology is very important for the further development of this area.

### Research hotspots and thematic evolution based on keyword analysis

The bibliometric analysis of the keyword distribution gives some important information about the main research topics and new trends in the H_2_S-biomaterial research field.

#### Material platforms: hydrogel-based delivery systems

For example, among all the keywords mentioned, the keyword “hydrogel” appeared as the third most frequent one and had significant links to “hydrogen sulfide” and “wound healing.” Therefore, hydrogels can be regarded as the most widely investigated material platform in the H_2_S-biomaterial research field because of their advantageous physical and chemical properties. Hydrogels are characterized by high water content and have many other beneficial characteristics, which make them promising materials for the treatment of chronic wounds.^18^,^26^ In addition to hydrogels, keywords associated with nanomaterials, such as “nanoparticle” and “electrospinning,” indicate the diversified designs of biomaterials. Electrospinning is an easy, inexpensive, and flexible process; various materials can be used in this process, making it the preferred technique for the mass production of nanofibers.^27^ Nanofibers fabricated by electrospinning have a high specific surface area and tunable porosity, making it possible for them to be used as drug delivery systems and mimics of the extracellular matrix.^28^,^29^ Metals and metal oxides are antibacterial in nature.^30^,^31^,^32^ Sulfur-containing metal nanoparticles, such as copper sulfide (CuS) and zinc sulfide (ZnS), can be synthesized to achieve localized and sustained H_2_S release.^30^,^31^,^32^

#### Wound types: diabetic, burn, and infected wounds

Of all the disease-related keywords, “diabetic wound healing” is the most notable research focus. This keyword has a high frequency, is located in the center of the keyword co-occurrence network, and belongs to the basic theme category. From the location of this topic on the map, we can infer that research in this area has a relatively mature system and plays a significant role. This research tendency is mainly attributed to the increasing incidence of diabetes worldwide and the nature of diabetic wounds, which include chronic inflammation, oxidative stress, poor angiogenesis, and repeated infection. The comprehensive regulatory effects of H_2_S on these factors are directly relevant to this issue.^19^,^33^ “Burn wound” belongs to the “niche theme” quadrant of the strategic thematic map, and this implies that despite having a well-established internal system, this topic has low centrality. This suggests that research on burn injuries has developed a certain understanding of the underlying mechanisms, but it has not yet become part of the mainstream, which focuses on diabetic wounds. “Infected wound” was less frequent and belonged to the emerging/declining themes quadrant, which suggests that this theme is either in the initial stage of exploration or has already been replaced by other themes.

#### Therapeutic mechanisms: anti-inflammatory, antibacterial, and angiogenesis effects

The keyword co-occurrence network analysis indicated that there are three main clusters of topics, namely “anti-inflammatory,” “antibacterial,” and “angiogenesis,” which form the core of H_2_S wound healing research. This finding supports, from a bibliometric perspective, the core biological mechanisms through which H_2_S promotes wound healing. Inflammation, infection, and poor angiogenesis are the main pathological conditions that impair wound healing, and H_2_S can address all three through multiple complementary mechanisms: inhibition of inflammation through ROS scavenging and inhibition of the nuclear factor-κB signaling pathway,^8^,^9^,^11^ inhibition of bacteria by disrupting bacterial membrane integrity,^12^,^13^ and stimulation of angiogenesis by regulating vascular endothelial growth factor expression through the phosphatidylinositol 3-kinase/Akt signaling pathway.^6^,^7^

Specifically, the appearance of the emerging keyword “bacterial ferroptosis” demonstrates growing interest in the role of H_2_S in antibacterial strategies beyond antibiotics. Current research shows that biomaterials containing iron sulfides and capable of releasing both iron ions and H_2_S may induce ferroptosis-like cell death in bacteria. However, the exact mechanism underlying the role of H_2_S in this process remains unknown and requires additional research.^26^,^34^ In conclusion, the multi-targeted activity of H_2_S is a central driving force behind the development of research in this area, while the evolution of keywords from “antibacterial” to “bacterial ferroptosis” shows that research on antibacterial mechanisms is progressing from the macroscopic level to the molecular level.

#### Hydrogen sulfide release strategies: pH-responsive systems, photothermal therapy, and multimodal therapy

The strategic theme map highlighted more closely related themes associated with intelligent H_2_S release, including “pH responsive,” “photothermal therapy,” and “multimodal therapy,” suggesting that the development of intelligent and controllable H_2_S delivery platforms has become a hot topic of research interest. The extensive application of pH-responsive systems is attributed to the acidic environment of inflamed and infected wounds. By taking advantage of this endogenous stimulus, H_2_S can be released in a timely manner without any external control. This is considered one of the most facile intelligent H_2_S release strategies to design.^35^ In contrast, photothermal therapy takes advantage of near-infrared light to induce local heat to exert antibacterial effects while inducing H_2_S release from thermoresponsive carriers for wound repair.^23^,^36^ With increasing research on intelligent H_2_S-releasing materials in recent years, multimodal therapeutic platforms have been extensively developed, integrating multiple strategies, including pH responsiveness, enzyme responsiveness, ROS responsiveness, and photothermal treatment, into one material system. For example, Hao et al.^37^ created a self-healing lysozyme hydrogel with multifunctionality through an enzyme-driven mechanism, in which glucose oxidase leads to the formation of an acidic microenvironment and the simultaneous release of H_2_S. Such multimodal systems may enable precise spatiotemporal regulation of H_2_S release and provide synergistic therapeutic effects.

Generally, the development of the field is progressing from single-stimulus-responsive systems toward multi-responsive intelligent systems, indicating a high degree of alignment between material design and the complexity of the pathological environment.

### Recent advances in hydrogen sulfide-based biomaterials for wound healing

H_2_S-based biomaterials for wound healing have evolved from simple sustained-release formulations into more sophisticated multifunctional treatment platforms. The range of H_2_S delivery platforms has expanded beyond traditional hydrogels through the introduction of electrospun fibers, microneedle patches, nanomaterials, and microenvironment-responsive biomaterials.

Hydrogels are the most common carriers used for H_2_S delivery due to their favorable properties, including sustained release, tunable physicochemical properties, and biocompatibility.^17^ Hydrogel-based H_2_S delivery systems can be categorized according to whether H_2_S donors are physically encapsulated, covalently conjugated, or released in response to the microenvironment.^38^,^39^ Stimuli-responsive systems rely on either endogenous stimuli (e.g., pH and ROS) or exogenous stimuli (e.g., temperature and light) to achieve on-demand release.^17^ A composite hydrogel composed of recombinant human collagen, carboxymethyl chitosan, and tannic acid containing an ROS-responsive H_2_S donor was recently developed by Yang et al.^18^ Elevated ROS levels in the oxidative microenvironment induce the on-demand release of H_2_S via the activation of the ROS-responsive H_2_S donor. In the case of pH-responsive delivery, Sun et al.^35^ fabricated an injectable polysaccharide hydrogel containing α-phase manganese sulfide (MnS) nanoparticles. Acid-labile Schiff-base cross-links control the release of the nanoparticles, while H_2_S is released on demand through a pH-dependent reaction. In another study, Xu et al.^26^ created a self-healing hydrogel with phycocyanin-functionalized FeS nanoparticles (FeS@PC NPs). The hyperthermia generated by the photothermal effect under 660 nm laser irradiation causes iron sulfide (FeS) degradation, resulting in H_2_S release and hydroxyl radical (·OH) generation via a Fenton reaction catalyzed by ferrous ion (Fe^2+^).

Apart from hydrogels, H_2_S delivery has recently been introduced into minimally invasive systems. Liu et al.^20^ developed a double-layered microneedle patch consisting of a keratin-based H_2_S donor and ciprofloxacin for efficient transdermal co-delivery, combining antibacterial effects with H_2_S-stimulated tissue repair. More broadly, microneedle-based platforms can improve transdermal delivery efficiency and patient adherence.^40^

The integration of nanotechnology has also emerged as a promising research direction. Yin et al.^34^ developed a single-component cascade nanozyme (FeS@Au), consisting of gold nanoclusters anchored on ferrous sulfide nanoparticles. This nanozyme reduces local glucose levels, triggers ferroptosis-like death in drug-resistant bacteria, and releases H_2_S, thereby increasing hypoxiainducible factor-1α expression and supporting angiogenesis. In a diabetic model, the system showed antibacterial activity while also enhancing angiogenesis and collagen deposition, ultimately accelerating wound closure.

A common feature of most of these platforms is their use of multimodal therapy to simultaneously target infection, inflammation, and impaired angiogenesis. A recent nano-hydrogel system loaded with glucose oxidase and ZnS/arginine-loaded MOF-818 composite nanoparticles (ZnS/Arg@MOF-818) combined glucose depletion, photothermal therapy, and synergistic nitric oxide/H_2_S gas therapy. It achieved complete bactericidal activity against multidrug-resistant bacteria in vitro and 98% wound closure in a diabetic model by day 9, together with markedly enhanced angiogenesis and collagen deposition.^22^

Overall, hydrogels remain the predominant carriers for H_2_S delivery, while microneedles and nanomaterials have broadened its potential applications. H_2_S delivery has also progressed toward precise, stimuli-responsive release, and treatment strategies have increasingly combined H_2_S with photothermal therapy, other gas-signaling molecules, and antimicrobial agents to regulate infection, inflammation, and angiogenesis simultaneously. These findings support the research frontiers revealed by our bibliometric analysis and suggest that smart biomaterials with multimodal functions and precise release control may play an important role in future clinical translation.

### Limitations

First, data collection ended on March 31, 2026, which meant that publications from the remainder of the year were not included. Consequently, the publication count for 2026 may not reflect the full annual output. Second, although total citation counts were presented for countries and institutions in **Tables 2** and **3**, these values were used as descriptive information rather than as the sole indicators of research influence. Citation relationships were also used to construct the journal citation network shown in **Figure 5C**. However, a large proportion of the included studies were published between 2024 and 2026 and had limited time to accumulate citations. This citation lag may have led to an underestimation of the influence of recently active countries, institutions, and journals and may also have affected the structure of the journal citation network.

### Future directions

From the results of the bibliometric analysis, some directions for future research may be established. First of all, more detailed studies should be conducted to identify the exact mechanisms of action of H_2_S during the wound healing process, especially mechanisms involving recently identified processes such as bacterial ferroptosis and interactions with other treatment methods. Secondly, an exciting area is the development of intelligent and multi-responsive biomaterials that combine multiple types of therapeutic effects, including photothermal therapy, nanozyme-catalyzed reactions, and on-demand H_2_S release.

## Conclusion

The current bibliometric analysis offers the first comprehensive review of the literature on H_2_S used in combination with biomaterials for wound healing from 2005 to March 31, 2026. During the entire investigation period, there was an overall positive trend in the number of publications. In addition, the largest number of publications was found in China. Hydrogels have been among the most common biomaterials in research. Keyword co-occurrence and thematic analysis showed that “H_2_S-hydrogel-wound healing” is a major research area in this field. Popular keywords included “anti-inflammatory,” “antibacterial,” “angiogenesis,” and “pH-responsive.” Analysis of thematic development indicated a gradual change from traditional scaffold approaches to multimodal therapy and new subjects, such as bacterial ferroptosis.

**Open Questions**

1. How can artificial intelligence (AI)-assisted literature retrieval and data mining reduce biases in bibliometric studies, and what validation standards are required?
2. Can AI-driven predictive modeling, informed by systematically retrieved literature, resolve the long-standing dose-response paradox of hydrogen sulfide (H_2_S) in chronic wound healing?
3. How should multimodal data (material properties, release kinetics, and *in vivo* outcomes), extracted via AI-assisted mining of heterogeneous sources, be integrated to facilitate clinical translation?

## Additional file:

**Additional file 1: Full search strategies**

**Web of Science Core Collection**

**Table:** 

No.	Search strategy
#1	TS=("hydrogen sulfide" OR "hydrogen sulphide" OR "H2S" OR "H_2_S" OR "sodium hydrosulfide" OR "sodium hydrosulphide" OR "NaHS" OR "GYY4137" OR "diallyl trisulfide" OR "DATS" OR "diallyl trisulphide" OR "AP39" OR "AP123" OR "sulfide salt" OR "H2S donor*" OR "H_2_S donor*" OR "hydrogen sulphide donor" OR "hydrogen sulfide donor" OR "sulfide-releasing" OR "sulphide-releasing" OR "H2S-releasing" OR "H_2_S-releasing")
#2	ALL=("biomaterial*" OR "hydrogel*" OR "scaffold*" OR "nanofiber*" OR "nanofibre*" OR "membrane*" OR "film" OR "membrane" OR "patch" OR "polymer*" OR "copolymer*" OR "liposome*" OR "nanoparticle*" OR "nanomaterial*" OR "nanocomposite*" OR "microsphere*" OR "microneedle*" OR "dressing*" OR "nanostructure*" OR "nanozyme*" OR "nano-antibacterial*" OR "nanoantibacterial*" OR "nanoplatform*" OR "nanocarrier*")
#3	ALL=("wound heal*" OR "wound repair" OR "tissue repair*" OR "tissue regenerat*" OR "skin regenerat*" OR "ulcer" OR "burn*" OR "tissue engineering" OR "regenerative medicine" OR "chronic wound*" OR "diabetic foot" OR "diabetic ulcer*" OR "cutaneous repair" OR "dermal regenerat*" OR "wound closure" OR "wound dress*" OR "wound regenerat*" OR "diabetic wound*" OR "infected wound*" OR "skin wound*" OR "cutaneous wound*" OR "dermal wound*" OR "implant-related infection*" OR "implant infection*" OR "prosthetic infection*" OR "biofilm infection*")
#4	#1 AND #2 AND #3

**Scopus**

**Table:** 

No.	Search strategy
#1	TITLE-ABS-KEY("hydrogen sulfide" OR "hydrogen sulphide" OR "H2S" OR "H_2_S" OR "sodium hydrosulfide" OR "sodium hydrosulphide" OR "NaHS" OR "GYY4137" OR "diallyl trisulfide" OR "DATS" OR "diallyl trisulphide" OR "AP39" OR "AP123" OR "sulfide salt" OR {H2S donor*} OR {H_2_S donor*} OR "hydrogen sulphide donor" OR "hydrogen sulfide donor" OR "sulfide-releasing" OR "sulphide-releasing" OR "H2S-releasing" OR "H_2_S-releasing")
#2	TITLE-ABS-KEY("biomaterial*" OR "hydrogel*" OR "scaffold*" OR "nanofiber*" OR "nanofibre*" OR "membrane*" OR "film" OR "patch" OR "polymer*" OR "copolymer*" OR "liposome*" OR "nanoparticle*" OR "nanomaterial*" OR "nanocomposite*" OR "microsphere*" OR "microneedle*" OR "dressing*" OR "nanostructure*" OR "nanozyme*" OR "nano-antibacterial*" OR "nanoantibacterial*" OR "nanoplatform*" OR "nanocarrier*")
#3	TITLE-ABS-KEY("wound heal*" OR "wound repair" OR "tissue repair*" OR "tissue regenerat*" OR "skin regenerat*" OR "ulcer" OR "burn*" OR "tissue engineering" OR "regenerative medicine" OR "chronic wound*" OR "diabetic foot" OR "diabetic ulcer*" OR "cutaneous repair" OR "dermal regenerat*" OR "wound closure" OR "wound dress*" OR "wound regenerat*" OR "diabetic wound*" OR "infected wound*" OR "skin wound*" OR "cutaneous wound*" OR "dermal wound*" OR "implant-related infection*" OR "implant infection*" OR "prosthetic infection*" OR "biofilm infection*")
#4	#1 AND #2 AND #3

**PubMed**

**Table:** 

No.	Search strategy
#1	("hydrogen sulfide"[tiab] OR "hydrogen sulphide"[tiab] OR "H2S"[tiab] OR "H_2_S"[tiab] OR "sodium hydrosulfide"[tiab] OR "sodium hydrosulphide"[tiab] OR "NaHS"[tiab] OR "GYY4137"[tiab] OR "diallyl trisulfide"[tiab] OR "DATS"[tiab] OR "diallyl trisulphide"[tiab] OR "AP39"[tiab] OR "AP123"[tiab] OR "sulfide salt"[tiab] OR H2S donor*[tiab] OR H_2_S donor*[tiab] OR "hydrogen sulphide donor"[tiab] OR "hydrogen sulfide donor"[tiab] OR "sulfide-releasing"[tiab] OR "sulphide-releasing"[tiab] OR "H2S-releasing"[tiab] OR "H_2_S-releasing"[tiab])
#2	(biomaterial*[tiab] OR hydrogel*[tiab] OR scaffold*[tiab] OR nanofiber*[tiab] OR nanofibre*[tiab] OR membrane*[tiab] OR film[tiab] OR patch[tiab] OR polymer*[tiab] OR copolymer*[tiab] OR liposome*[tiab] OR nanoparticle*[tiab] OR nanomaterial*[tiab] OR nanocomposite*[tiab] OR microsphere*[tiab] OR microneedle*[tiab] OR dressing*[tiab] OR nanostructure*[tiab] OR nanozyme*[tiab] OR nano-antibacterial*[tiab] OR nanoantibacterial*[tiab] OR nanoplatform*[tiab] OR nanocarrier*[tiab])
#3	(wound heal*[tiab] OR "wound repair"[tiab] OR tissue repair*[tiab] OR tissue regenerat*[tiab] OR skin regenerat*[tiab] OR ulcer[tiab] OR burn*[tiab] OR "tissue engineering"[tiab] OR "regenerative medicine"[tiab] OR chronic wound*[tiab] OR "diabetic foot"[tiab] OR diabetic ulcer*[tiab] OR "cutaneous repair"[tiab] OR dermal regenerat*[tiab] OR "wound closure"[tiab] OR wound dress*[tiab] OR wound regenerat*[tiab] OR diabetic wound*[tiab] OR infected wound*[tiab] OR skin wound*[tiab] OR cutaneous wound*[tiab] OR dermal wound*[tiab] OR implant-related infection*[tiab] OR implant infection*[tiab] OR prosthetic infection*[tiab] OR biofilm infection*[tiab])
#4	#1 AND #2 AND #3

## Data Availability

*All data generated or analyzed in this study are included in this published article and its additional file.*
